# Análisis espacial de las concentraciones de PM_25_ en Bogotá según los valores de las guías de la calidad del aire de la Organización Mundial de la Salud para enfermedades cardiopulmonares, 2014-2015

**DOI:** 10.7705/biomedica.4719

**Published:** 2020-03-30

**Authors:** Laura Andrea Rodríguez-Camargo, Ronal Jackson Sierra-Parada, Luis Camilo Blanco-Becerra

**Affiliations:** 1 Facultad de Ingeniería Ambiental, Universidad Santo Tomás, Bogotá, D.C, Colombia Universidad Santo Tomás Facultad de Ingeniería Ambiental Universidad Santo Tomás BogotáD.C Colombia; 2 Maestría en Salud Pública, Universidad Santo Tomás, Bogotá, D.C, Colombia Universidad Santo Tomás Universidad Santo Tomás BogotáD.C Colombia

**Keywords:** sistemas de información geográfica, material particulado, enfermedad cardiopulmonar, estudios ecológicos, poblaciones vulnerables, Geographic information system, particulate matter, pulmonary heart disease, ecological studies, vulnerable populations

## Abstract

**Introducción.:**

La Organización Mundial de la Salud señala que tres millones de muertes al año por enfermedades cardiopulmonares están relacionadas con la exposición a la contaminación del aire.

**Objetivo.:**

Estimar las superficies de concentración de partículas en suspensión de menos de 2,5 pm *(Particulate Matter,* PM_25_) en Bogotá entre el 2014 y el 2015, clasificándolas según las guías de calidad del aire de la Organización Mundial de la Salud para enfermedades cardiopulmonares.

**Materiales y métodos.:**

Se hizo un estudio ecológico mediante técnicas geoestadísticas. Se calcularon los promedios de PM_25_ en lapsos de seis horas a lo largo del día en cuatro franjas horarias. Las concentraciones se clasificaron según los valores diarios y anuales de las guías de calidad del aire de la OMS.

**Resultados.:**

La localidad de Kennedy presentó las mayores concentraciones de PM_25_ en todas las franjas horarias. Los valores registrados en esta zona y clasificados según las guías diarias y anuales de calidad del aire, evidenciaron que la localidad presentaría un incremento de 1,2 % en la mortalidad cardiopulmonar en el corto plazo y de 9 % en el largo plazo.

**Conclusión.:**

Las franjas horarias de las 0:00 a las 6:00 h y de las 12:00 a las 18:00 h, cumplieron con el valor anual de las guías de calidad del aire de 10 µg/m^3^ en una parte de la zona oriental de la ciudad. En el resto de la ciudad, en las franjas horarias de las 6:00 h a las 12:00 h y de las 18:00 h a las 24:00 h se registraron valores que cumplían los objetivos intermedios 2 y 3, lo que representa incrementos de 9 y 3 % en la mortalidad cardiopulmonar, respectivamente.

## Abstract (en)

**Introduction:**

The World Health Organization (WHO) points out that 3 million deaths per year caused by cardiopulmonary diseases are related to exposure to air pollution.

**Objective:**

To estimate areas of concentration of PM_2.5_ in Bogotá according to the WHO Air Quality Guidelines (AQG) for cardiopulmonary diseases during the period 2014-2015.

**Materials and methods:**

We conducted an ecological study with geostatistical techniques. We calculated the PM_2.5_ averages for six hour-periods distributed throughout the day in four time slots, which were classified according to daily and annual WHO AQG.

**Results:**

The locality of Kennedy presented the highest concentrations of PM_25_ in all time slots. The values registered in this area classified within the daily and annual AQG showed that the locality would present an increase of 1.2% and 9% in cardiopulmonary mortality in the short and long term, respectively.

**Conclusion:**

The time slots from 0:00 to 6:00 h y from 12:00 to 18:00 h met the annual AQG value of 10 µg/m^3^ in a part of the eastern zone of the city; in the rest of the city, in these same time slots, intermediate objectives 2 and 3 were met, which means increases by 9% and 3% in the cardiopulmonary mortality according to the AQG, respectively.

Nueve de cada diez personas respiran aire con altos niveles de contaminación. En el 2012, la Organización Mundial de la Salud (OMS) señaló que 6,5 millones de muertes (11,6 % de todas las muertes en el mundo) estuvieron relacionadas con la contaminación del aire en interiores y exteriores, y que el 94 % de ellas se debieron a enfermedades cardiovasculares, accidentes cerebrovasculares, enfermedad pulmonar obstructiva crónica y cáncer de pulmón; además, el riesgo de infecciones respiratorias agudas aumentó. El 90 % de las muertes por aire contaminado se produjo en los países de ingresos bajos y medios, y casi dos de cada tres se registraron en las regiones de Asia suroriental y del Pacífico occidental de la OMS ^1^. El crecimiento urbanístico y poblacional, sumado a las diferentes actividades económicas e industriales, produce emisiones contaminantes del aire, como el ozono (O_3_), el dióxido de nitrógeno (NO_2_) y el dióxido de azufre (SO_2_), y de material en suspensión *(Particulate Matter,* PM), siendo este último uno de los de mayor interés debido a las concentraciones promedio diarias y anuales registradas en diferentes ciudades del mundo ^2^^-^^5^.

Bogotá es una de las ciudades con mayores problemas por calidad del aire, principalmente por el material en suspensión generado, principalmente, por fuentes móviles y por el sector industrial ^6^. El Instituto de Hidrología, Meteorología y Estudios Ambientales (IDEAM) estableció que, entre los años 2011 y 2015, las PM_10_ y PM_2 5_ en las estaciones de Kennedy y Carvajal registraron los promedios anuales más altos del país donde, aproximadamente, el 20 % reportó valores de PM_10_ de más de 100 µg/m^3^ y que cerca del 15 % de los datos correspondía a concentraciones superiores a 40 µg/m^3^ de PM_2,5_, es decir que el índice de calidad del aire correspondía a la categoría de "dañina para la salud" ^7^.

Los efectos en la salud humana causados por el material en suspensión se deben principalmente a su diámetro, que permite su entrada a las vías respiratorias, donde producen daños en los tejidos y órganos e, incluso, el transporte de virus y bacterias ^8^.

La exposición a PM_2,5_ se ha relacionado con enfermedades respiratorias, cáncer pulmonar, silicosis y afectaciones del sistema cardiovascular, entre otras. Estas enfermedades se deben a que, además de ser un tipo de partícula respirable, las PM_25_ son una mezcla de compuestos orgánicos e inorgánicos con una composición química muy variada que incluye metales, como el calcio, el zinc, el silicio, el plomo, el hierro y el cadmio, y por compuestos como el azufre, el carbono y el nitrógeno ^8^^,^^9^.

A nivel mundial se estima que las partículas finas causan alrededor del 8 % de las muertes por cáncer de pulmón, el 5 % por causas cardiopulmonares y el 3% por infecciones respiratorias. En países como Egipto, Indonesia, India, China y Tailandia, se excede más de tres veces el valor de PM_10_ propuesto en las guías de la calidad del aire *(Air Quality Guidelines)* de la OMS ^10^. En los estudios realizados en más de 30 ciudades del mundo, se ha comprobado la relación de la mortalidad y la morbilidad total, cerebrovascular y respiratoria, con los niveles de PM_10_ y de PM_25_, especialmente en las ciudades de clima seco; en estos estudios se han determinado mecanismos de daño inflamatorio, de estrés oxidativo y alteraciones del sistema nervioso ^11^.

La contaminación del aire en Colombia genera efectos negativos en el medio ambiente y en la salud humana, lo cual se ratifica en los antecedentes que sustentan la creación de la política integral de salud ambiental del país ^12^^,^^13^. Según el Departamento Nacional de Planeación, los costos en salud atribuibles a la contaminación del aire urbano en Colombia ascendieron a cerca de COP$ 15,4 billones de pesos (1,93 % del PIB del país) en el 2015 y estuvieron asociados con 10.500 muertes y 67,8 millones de registros de síntomas y enfermedades. En Bogotá, 3.219 de las muertes (10,5 %) se atribuyó a la contaminación del aire urbano, lo cual generó costos estimados en COP$ 4,2 billones (2,5 % del PIB de la ciudad) ^14^.

En este contexto, el objetivo del presente trabajo fue estimar superficies de concentración de PM_25_ en Bogotá entre el 2014 y el 2015, mediante técnicas geoestadísticas y clasificarlas según los valores diarios y anuales de las guías de calidad del aire de la OMS para enfermedades cardiopulmonares.

## Materiales y métodos

### Tipo de estudio

Se hizo un estudio ecológico de análisis espacial mediante técnicas geoestadísticas de interpolación *(kriging)* y de distancia inversa ponderada *(Inverse Distance Weighting,* IDW) para describir y analizar las variaciones geográficas de los factores ambientales de riesgo ^15^, en este caso, las concentraciones de PM_25_. Se seleccionó la ciudad de Bogotá, la cual se ubica a 2.640 metros sobre el nivel del mar y se caracteriza por un clima moderadamente frío, con cerca de 14 °C en los periodos de lluvia de marzo a mayo y de septiembre a noviembre ^16^; la ciudad se divide en 20 unidades administrativas (localidades) y tiene una población de 7'878.783 habitantes ^17^.

### Información meteorológica y de contaminantes del aire

Se obtuvo información de las concentraciones de PM_10_y PM_25_en las diversas franjas horarias en las estaciones de Carvajal, Centro de Alto Rendimiento, Kennedy, Ferias, Ministerio de Ambiente, Guaymaral, San Cristóbal, Usaquén, Tunal y Suba, pertenecientes a la Red de Monitoreo de Calidad del Aire de Bogotá durante el 2014 y el 2015.

El periodo de análisis y las estaciones se seleccionaron en función de la disponibilidad de registros horarios de PM_10_y PM_25_ (criterio de suficiencia de los datos de 75 %) y su continuidad en la medición durante los años de estudio.

**Figura 1:**
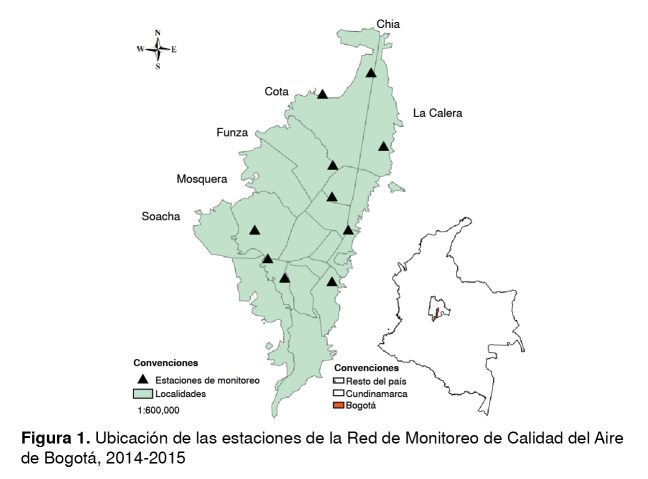
Ubicación de las estaciones de la Red de Monitoreo de Calidad del Aire de Bogotá, 2014-2015

La distribución y la ubicación de las estaciones de la Red de Monitoreo utilizadas para elaborar los mapas de dispersión de PM_25_ se aprecian en la figura 1. Las estaciones de la parte urbana de la ciudad se encuentran en las zonas occidental, central y norte, razón por la cual los mapas del análisis final evidenciaron una mejor predicción de las concentraciones de PM_25_ en estos sitios.

### *Análisis de las concentraciones de PM*_*10*_ *y PM*_*2B*_

Se hizo un análisis estadístico descriptivo de los datos horarios de PM_10_ y PM_25_. Para solucionar el problema de los datos faltantes de PM_10_, se hicieron gráficos de secuencia y, posteriormente, las correspondientes imputaciones mediante las técnicas de media de series (estima la media absoluta de todos los datos y así se completan los valores faltantes) ^18^ y de media de puntos cercanos (calcula el promedio de los puntos más cercanos al que adolece de información) ^18^.

Estos cálculos se hicieron en las 10 estaciones que disponían como mínimo del 75 % de los datos horarios; se utilizaron estos dos métodos porque la tendencia de los datos variaba según la estación y el periodo, por lo que en cada caso se recurrió a la técnica que mejor se ajustaba al comportamiento de los datos evidenciado en las gráficas de secuencia. Posteriormente, mediante la relación PM_25_/PM_10_, se obtuvieron los datos faltantes de PM_25_ en los registros horarios en que no se disponía de su concentración. En Colombia, en los estudios de Rojas ^19^, Echeverri ^20^, Blanco ^21^ y Larsen ^22^, se ha comprobado que dicha relación es una herramienta para estimar las concentraciones de PM_25_ a partir de los datos existentes de PM_10_.

Luego de obtener las concentraciones horarias de PM_2,5_, se generaron mapas de dispersión con el programa SIG. Para ello, fue necesario reorganizar los datos obteniendo promedios mensuales y anuales por estación, separándolos en cuatro franjas horarias (franja a: 0:00 a 6:00 h; franja b: 6:00 a 12:00 h; franja c: 12:00 a 18:00 h; y franja d: 18:00 a 24:00 h), con el fin de analizar las concentraciones y su comportamiento, las cuales cambian debido a las variables meteorológicas y a las fuentes móviles y fijas responsables de las emisiones, según los análisis llevados a cabo por la Red de Monitoreo de Calidad del Aire de Bogotá ^23^.

Para generar los mapas, se creó una base geográfica de datos que permitiera analizar tanto la información sobre los PM_2,5_ como la posición de las estaciones utilizadas en el estudio. Posteriormente, los datos georreferenciados se utilizaron en el análisis geoestadístico para recrear una superficie de predicción a partir de los puntos muestreados.

Dicho análisis se fundamentó en la evaluación de los datos con el método determinístico de distancia inversa ponderada, con el cual se estimaron las predicciones en las regiones circundantes a las estaciones de monitoreo utilizadas ^24^. Además, con la técnica de interpolación *(kriging)* se exploraron las relaciones estadísticas entre los datos de las estaciones, y se obtuvo una correlación espacial de hasta el 85 % en la superficie de predicción generada ^25^.

Para el análisis exploratorio de los datos se utilizó la llamada densidad de Kernel, herramienta de análisis estadístico empleada para representar la frecuencia de aparición de los datos o una variable visual, calculando la densidad en los mapas de un área de búsqueda circular, con el fin de determinar la distancia para expandir los valores alrededor de cada ubicación ^26^. Con base en el análisis de densidad de Kernel, se seleccionó el método de estadísticas geográficas de interpolación *(kriging)* para generar las superficies de concentración.

Por último, los niveles de PM_2,5_ por franja horaria se clasificaron según los valores de referencia recomendados por la OMS para las enfermedades cardiopulmonares. Las concentraciones provenientes de la base de datos de la Red de Monitoreo de Calidad del Aire de Bogotá se catalogaron dentro de los valores de los objetivos intermedios y los de las guías de calidad del aire de la OMS ^27^ en cuanto al promedio diario (cuadro 1) y anual (cuadro 2) de PM_25_.

**Cuadro 1:** Objetivos intermedios de PM_25_ y guías de la calidad del aire de la OMS: concentración media diaria

Objetivos	µg/m^3^	Fundamento del nivel
OI-1	75	Incremento de alrededor del 5 % de la mortalidad a corto plazo sobre el valor de las guías de calidad del aire
OI-2	50	Incremento de alrededor del 2,5 % de la mortalidad a corto plazo sobre el valor de las guías de calidad del aire
OI-3	37,5	Incremento de alrededor del 1,2% de la mortalidad a corto plazo sobre el valor de las guías de calidad del aire
GCA	25	Basado en la relación entre los niveles de partículas en suspensión de 24 horas y anuales

OI: objetivo intermedio; GCA: guía de calidad del aire de la OMS

**Cuadro 2:** Objetivos intermedios de PM_25_ y guías de calidad del aire sugeridos por la OMS: concentración media anual

Objetivos	µg/m^3^	Fundamento del nivel
OI-1	35	Estos niveles están asociados con un riesgo de mortalidad a largo plazo de alrededor de 15 % más que con el nivel de la guías de calidad del aire.
OI-2	25	Además de otros beneficios para la salud, estos niveles reducen el riesgo de mortalidad prematura en 6 % aproximadamente (2-11 %) en comparación con el nivel del OI-1.
OI-3	15	Además de otros beneficios para la salud, estos niveles reducen el riesgo de mortalidad en un 6 % (2-11 %) aproximadamente en comparación con el nivel del OI-2.
GCA	10	Estos son los niveles más bajos con los cuales se ha demostrado con más de un 95 % de confianza, que la mortalidad total, cardiopulmonar y por cáncer de pulmón, aumenta por la exposición prolongada al PM2,5.

OI: objetivo intermedio; GCA: guía de calidad del aire de la OMS

## Resultados

### *Comportamiento de las concentraciones de PM*_*10*_

Las concentraciones medias por franja horaria registradas en las estaciones de la Red de Monitoreo de Calidad del Aire de Bogotá variaron entre 37,2 µg/m^3^ (localidad de Usaquén) y 91,1 µg/m^3^ (localidad de Kennedy), donde las máximas concentraciones medias horarias se registraron en las estaciones de Carvajal y Kennedy (91,1 y 71,2 µg/m^3^ en el 2014, y 86,4 y 66,3 µg/m^3^ en el 2015), las dos en la localidad de Kennedy. El porcentaje de datos horarios disponibles evidenció que todas las estaciones de la Red de Monitoreo de Calidad del Aire de Bogotá presentaron porcentajes por encima del 75 %, cumpliendo así con el criterio de suficiencia del estudio, lo que permitió hacer la posterior imputación de datos faltantes.

En general, las estaciones de Carvajal y Kennedy excedieron el valor promedio diario establecido en la normatividad colombiana (Resolución 2254 de 2017) durante el 70 % (n=510) y el 36 % (n=262) de los días del periodo de estudio, respectivamente, en tanto que el valor promedio diario establecido por la OMS se excedió en estas dos estaciones en el 92 % (n=670) y el 75 % (n=548) de los días, respectivamente. En cuanto al promedio anual, solamente las estaciones de Carvajal y Kennedy sobrepasaron el valor de 50 µg/m^3^ establecido en la Resolución 2254 de 2017, pero en ninguna se cumplió el valor sugerido por la OMS (20 µg/m^3^).

### *Comportamiento de las concentraciones de PM*_*2B*_

Las concentraciones medias horarias registradas en las estaciones de la Red de Monitoreo de Calidad del Aire de Bogotá se ubicaron entre los 8,9 µg/ m^3^ (localidad de San Cristóbal) y los 33,6 µg/m^3^ (localidad de Kennedy), con las máximas concentraciones medias horarias en las estaciones de Carvajal y Kennedy (33,6 y 32,6 µg/m^3^ en el 2014, y 30,7 y 27 µg/m^3^ en el 2015), las dos en la localidad de Kennedy. Los valores máximos horarios se ubicaron en el rango de 64 µg/m^3^ (localidad de San Cristóbal) y 170 µg/m^3^ (localidad de Tunjuelito). La mayoría de las estaciones contaban con más del 74 % de los datos horarios de PM_2,5_, los cuales se complementaron haciendo uso de la relación PM_2,5_/PM_10_ durante la hora anterior o posterior a aquella en la que se disponía de los datos de los dos valores (cuadro 3).

**Cuadro 3:** Concentración horaria de PM_2,5_ en la Red de Monitoreo de Calidad del Aire de Bogotá, 2014 y 2015

Localidad	Estación	Media µg/m^3^		DE		Máx. µg/m^3^		% de datos disponibles	
		2014	2015	2014	2015	2014	2015	2014	2015
Kennedy	Carvajal	33,6	30,7	88,6	13,8	152	149	87,6	94,5
Barrios Unidos	Centro de Alto Rendimiento	22,5	15,9	83,3	11,4	118	132	77,2	92,3
Suba	Guaymaral	19,2	16,1	44,2	10,9	90	118	21,9	94,4
Kennedy	Kennedy	32,6	27,0	89,8	15,1	159	135	89,6	98,3
Engativá	Ferias	20,6	17,0	78,2	12,4	143	96	68,0	74,3
Santa Fe	Ministerio del Medio Ambiente	17,3	14,7	81,1	12,9	166	311	73,3	97,6
San Cristóbal	San Cristóbal	10,2	8,9	73,9	6,9	64	72	59,2	89,3
Suba	Suba	22,5	21,6	83,1	12,7	124	105	77,2	92,1
Tunjuelito	Tunal	24,7	21,3	90,2	14,4	170	114	90,1	95,0
Usaquén	Usaquén	15,0	12,9	82,5	9,7	77	96	76,6	86,0

DE: desviación estándar; Máx.: máximo

Las estaciones de Carvajal y Kennedy sobrepasaron la concentración promedio diaria establecida en la Resolución 2254 de 2017 durante el 26 % (n=192) y el 22 % (n=159) de los días, respectivamente, en tanto que el valor promedio diario establecido por la OMS se excedió en las dos estaciones en el 80 % (n=585) y el 59 % (n=428) de los días, respectivamente. En cuanto al promedio anual, solamente las estaciones Carvajal y Kennedy sobrepasaron el valor de 25 µg/m^3^ establecido en la Resolución 2254 de 2017 y ninguna cumplió el valor sugerido por la OMS (10 µg/m^3^).

### *Comportamiento de las concentraciones de PM*_*25*_ *por franjas horarias*

Se calcularon los promedios por franjas (seis horas) en los años de estudio para conocer y analizar el comportamiento del PM_2 5_ en diferentes horas del día, ya que existen momentos con mayores concentraciones debido a las diferentes dinámicas sociales y económicas de la ciudad ^28^.

Los cálculos evidenciaron que los niveles promedio más altos se dieron en la franja b (6:00 a 12:00 h), con valores de 25,7 µg/m^3^ en el 2014 y de 21,5 µg/ m^3^ en el 2015, seguida por la franja d (18:00 a 24:00 h), con valores de 22,4 µg/m^3^ en el 2014 y de 19,5 µg/m^3^ en el 2015. Estas franjas abarcan las horas pico del tráfico en la ciudad, es decir que en estas horas se presentan los mayores niveles de inmisión debidos a la actividad industrial y comercial, así como el desplazamiento masivo de ciudadanos por toda la ciudad. En la franja a (0:00 a 6:00 h) se registró un promedio de seis horas con 18,9 µg/m^3^ en el 2014 y con 16,9 µg/m^3^ en el 2015, en tanto que en la franja c (12:00 a 18:00 h) hubo concentraciones de 19,8 µg/m^3^ en el 2014 y de 16,9 µg/m^3^ en el 2015.

Al clasificar los valores de los promedios de seis horas por franja según los valores de referencia diarios y anuales sugeridos en las guías de la OMS, se estableció que en el promedio de 24 horas no se excedió la concentración de dichas guías de 25 µg/m^3^, en tanto que el valor anual de 10 µg/m^3^ se superó en todas las franjas.

Como ya se mencionó, la franja b presentó los mayores niveles promedio de PM_25_, principalmente en las estaciones de Kennedy y Carvajal (39,4 y 38,3 µg/m^3^ en el 2014, y 32,8 y 32,5 µg/m^3^ en el 2015), en tanto que la estación de San Cristóbal reportó la menor concentración promedio (11,87 µg/m^3^ en el 2014 y 10,81 µg/m^3^ en el 2015) (cuadro 4).

**Cuadro 4:** Concentración promedio en seis horas de PM_25_ en la franja b de la Red de Monitoreo de Calidad del Aire de Bogotá, 2014 y 2015

Localidad	Estación	Media µg/m^3^		DE		Máx. µg/m^3^	
		2014	2015	2014	2015	2014	2015
Kennedy	Carvajal	38,3	32,5	18,9	15,3	152,3	149,9
Barrios Unidos	Centro de Alto Rendimiento	24,2	19,8	16,2	15,3	118,4	103,8
Suba	Guaymaral	18,9	15,8	14,2	12,4	87,5	82,3
Kennedy	Kennedy	39,4	32,83	20,9	19,1	125,8	120,5
Engativá	Ferias	22,2	19,9	14,4	13,9	96,1	96,5
San Cristóbal	San Cristóbal	11,9	10,8	9,3	7,8	64,2	61,4
Suba	Suba	21,8	21,8	14,7	14,4	86,0	104,0
Tunjuelito	Tunal	28,2	24,6	21,4	17,8	170,2	114,0
Usaquén	Usaquén	16,4	15,2	12,5	11,2	77,8	84,00
Santa Fe	Ministerio del Medio Ambiente	22,7	20,1	16,3	15,0	166,5	95,00
Promedio franja		25,7	21,5			170,2	149,9

DE: desviación estándar; Máx.: máximo

### *Mapas de concentración de PM*_*2 5*_ *en función de las guías de calidad del aire de la OMS para enfermedades cardiopulmonares*

Los promedios por franja obtenidos de la Red de Monitoreo de Calidad del Aire de Bogotá se clasificaron según los promedios diario y anual sugeridos en las guías de la OMS. Se elaboraron mapas en los cuales se definieron entre 2 y 4 categorías, y donde el número 1 representaba los valores de concentración más bajos y, el 4, los más altos, según los objetivos intermedios y los establecidos para el promedio diario y anual por la OMS (cuadros 1 y 2).

### *Comportamiento de las concentraciones de PM*_*2,5*_ *en función de los valores diarios de las guías de la OMS*

Las concentraciones del promedio de seis horas en las franjas no superaron los 38 µg/m^3^ y se ubicaron entre los valores de las guías de la OMS y el objetivo intermedio 3, es decir, entre los niveles más bajos en que se ha comprobado que la mortalidad cardiopulmonar aumenta por exposiciones crónicas a contaminantes en 1,2 % en un periodo de exposición corto ^27^ (cuadro 1).

**Figura 2:**
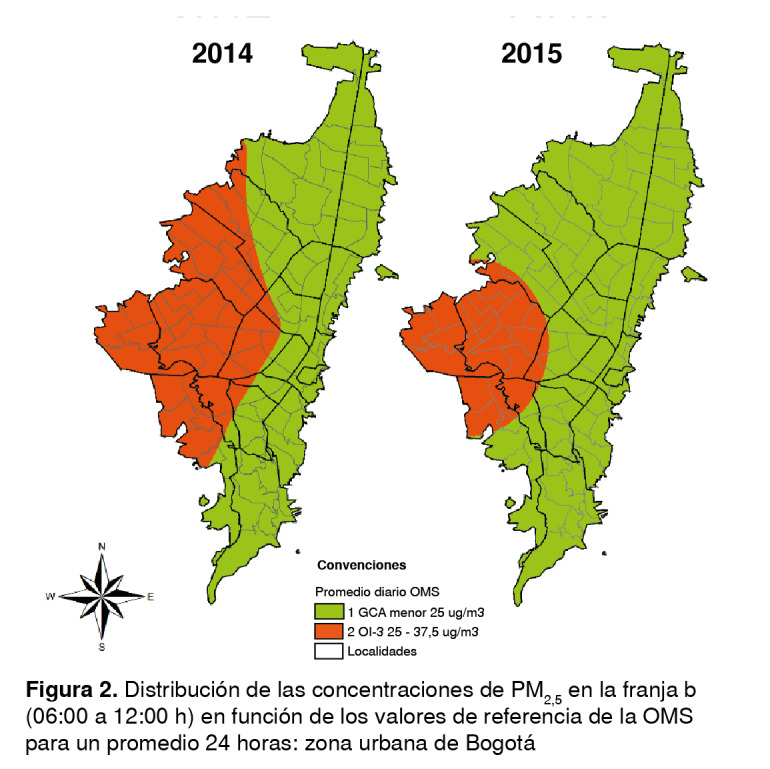
Distribución de las concentraciones de PM_25_ en la franja b

En el 2014, la franja a presentó su mayor concentración y se ubicó en el objetivo intermedio 3 en la localidad de Kennedy; el resto de la ciudad tuvo niveles bajos que cumplieron con los valores de las guías. Las franjas b y d en ese año registraron la mayor dispersión en el occidente, afectando casi a la mitad de la ciudad, específicamente las localidades de Bosa, Kennedy, Fontibón, Tunjuelito, Puente Aranda, Suba, Engativá, Teusaquillo, Ciudad Bolívar, Rafael Uribe Uribe y Antonio Nariño (figuras 2 y 3). En la franja c, la dispersión disminuyó notablemente, sin embargo, siguieron presentándose los mayores niveles de inmisión en la zona occidental que afectaron parcialmente a las localidades de Bosa, Kennedy, Tunjuelito y Ciudad Bolívar.

**Figura 3:**
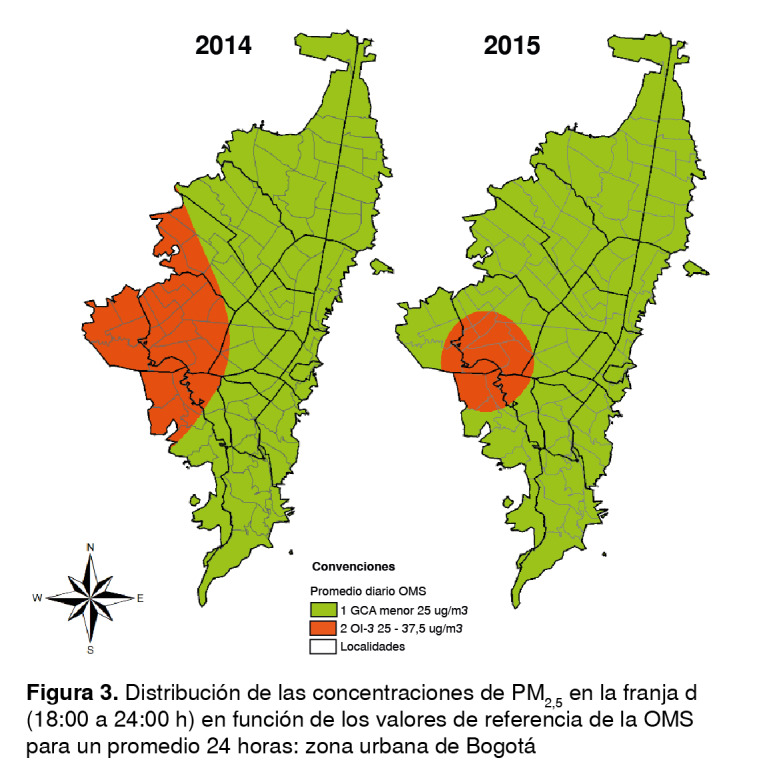
Distribución de las concentraciones de PM_25_ en la franja d (06:00 a 12:00 h) en función de los valores de referencia de la OMS (18:00 a 24:00 h) en función de los valores de referencia de la OMS para un promedio 24 horas: zona urbana de Bogotá para un promedio 24 horas: zona urbana de Bogotá

En el 2015, la franja a presentó tan solo un punto en el objetivo intermedio 3 en las unidades de planeación zonal de Corabastos y Kennedy Central (localidad de Kennedy), en tanto que en el resto de la ciudad se cumplieron los valores de las guías, aunque con una gran dispersión en el occidente, especialmente en la franja b, afectando nuevamente a toda la localidad de Kennedy y Bosa, parte de Ciudad Bolívar, Tunjuelito, Puente Aranda y Fontibón (figura 2). La franja c tan solo tuvo un punto con concentraciones ubicadas en el objetivo intermedio 3, en la unidad de planeación zonal de Carvajal, pero el resto de la ciudad cumplió con los valores de las guías; por último, en la franja d volvió a aumentar la dispersión en el occidente, pero en menor medida que en la franja b, afectando aproximadamente a 15 unidades de planeación zonal de esta zona de la ciudad; el resto cumplió con los valores de las guías (figura 3).

### *Comportamiento de las concentraciones de PM*_*2B*_ *en función de los valores anuales de las guías de la OMS*

En el 2014, en la franja a, la mayor parte de la ciudad (norte, sur, centro y una parte de occidente) se ubicó en el objetivo intermedio 2, con concentraciones de alrededor de 25 µg/m^3^, lo que aumentó el riesgo de mortalidad en 9 % tomando como base el valor de las guías (cuadro 2). La zona oriental de la capital se ubicó en el objetivo intermedio 3 y en los valores de las guías.

**Figura 4:**
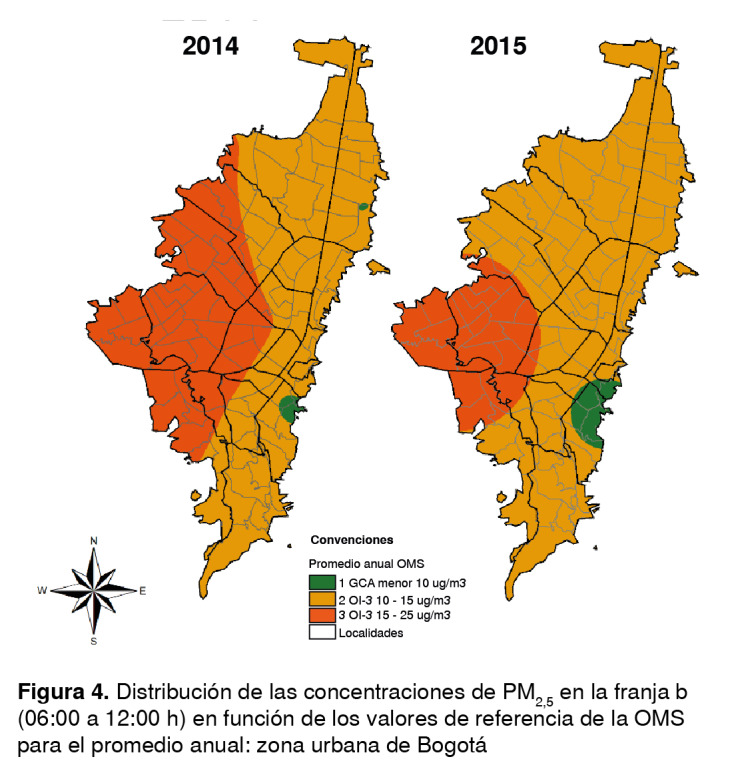
Distribución de las concentraciones de PM_25_ en la franja b

En la franja b, se observó una gran dispersión de las concentraciones alrededor de los 25 µg/m^3^ en la zona occidental, abarcando por completo las localidades de Bosa, Fontibón, Kennedy, Puente Aranda y, parcialmente, a Engativá, Antonio Nariño, Rafael Uribe Uribe, Tunjuelito y Ciudad Bolívar (figura 4). El resto de la ciudad se ubicó en el objetivo intermedio 3 y tan solo la parte norte de la unidad de planeación zonal de Usaquén y la parte sur de la unidad de planeación zonal de Los Cedros (localidad de Usaquén), así como la unidad de planeación zonal Lourdes (localidad de Santa Fe) y parte de las unidades de planeación zonal de Sosiego, 20 de Julio y San Blas (localidad de San Cristóbal) cumplieron con las guías, lo que indica que las concentraciones aumentaron en la parte oriental de la ciudad comparada con la franja a, en la cual se cumplió con los valores de las guías.

**Figura 5:**
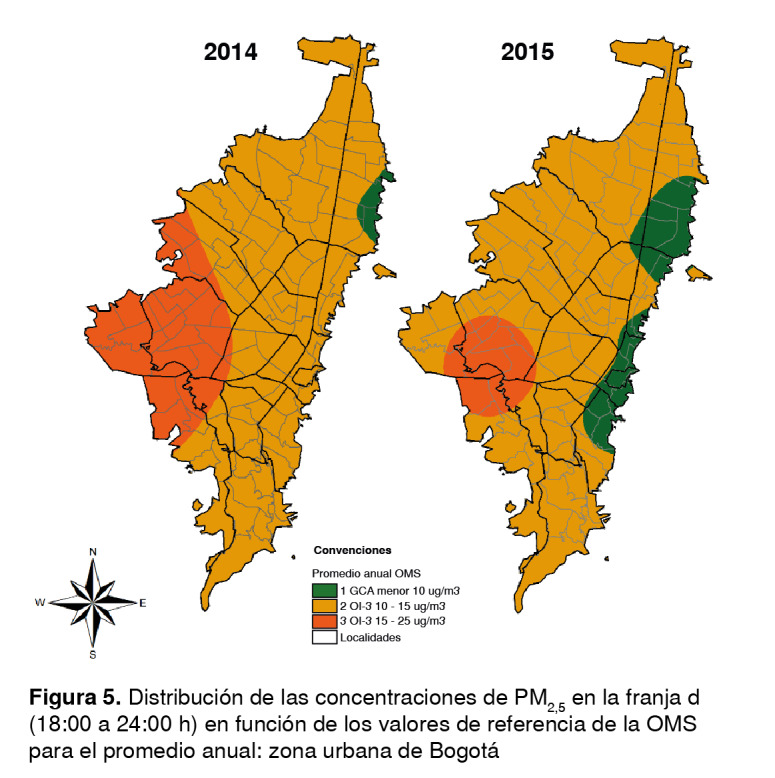
Distribución de las concentraciones de PM_25_ en la franja d (06:00 a 12:00 h) en función de los valores de referencia de la OMS (18:00 a 24:00 h) en función de los valores de referencia de la OMS para el promedio anual: zona urbana de Bogotá para el promedio anual: zona urbana de Bogotá

En la franja c decrecieron las concentraciones en el norte de la ciudad y se cumplió con los valores de las guías, específicamente en las localidades de Suba y Usaquén; en tanto que en el suroriente, las localidades de San Cristóbal y Santa Fe se ubicaron en el objetivo intermedio 3. En la parte occidental, los valores aumentaron y llegaron al objetivo intermedio 1, afectando de manera parcial a las localidades de Kennedy, Bosa, Tunjuelito y San Cristóbal. El resto de la ciudad se ubicó en el objetivo intermedio 2. Por último, en la franja d, las concentraciones disminuyeron, pero aumentó su dispersión en el occidente; así, en el resto de la ciudad se obtuvieron valores de alrededor de 15 µg/m^3^ y tan solo una mínima parte de la localidad de Usaquén cumplió con las guías, es decir, los niveles más bajos con los cuales se ha demostrado que la mortalidad total, cardiopulmonar y por cáncer de pulmón aumenta como reacción a la exposición prolongada al PM_25_^27^ (figura 5).

En el 2015, en la franja a la mayor parte de la ciudad se ubicó en el objetivo intermedio 2; en la zona oriental se registraron bajas concentraciones, ubicándose también en el objetivo intermedio 2, y en la zona norte de la localidad de San Cristóbal se cumplió con los valores de las guías. En la franja b, los valores disminuyeron en la mayor parte de la capital, ubicándose en el objetivo intermedio 3; en la localidad de San Cristóbal se mantuvo el cumplimiento de las guías, en tanto que en el occidente de la ciudad se registró una gran dispersión y valores aproximados de 25 µg/m^3^ (objetivo intermedio 2) (figura 4).

Por otro lado, comparadas con las del 2014, las concentraciones en la franja c continuaron disminuyendo con registros entre los 10 y 15 µg/m^3^ en toda la ciudad; en esta franja, el oriente de la ciudad cumplió con los valores de las guías y en el resto se ubicó en el objetivo intermedio 3. Por último, en la franja d, las concentraciones aumentaron nuevamente, principalmente en el occidente, ubicándose nuevamente en el objetivo intermedio 2 y afectando parte de las localidades de Kennedy, Bosa, Ciudad Bolívar, Tunjuelito y Puente Aranda (figura 5).

Teniendo en cuenta que gran parte de la ciudad presentó concentraciones de PM_25_ cercanas a los 37 µg/m^3^, se estableció que una proporción relevante de ciudadanos estuvo expuesta a estos niveles en franjas horarias que van de las 6:00 a 12:00 h y de las 18:00 a 24:00 h, aunque debe precisarse que no solo se vio afectada la población residente en estas áreas, sino la población flotante que trabaja, estudia o se moviliza con frecuencia en estas zonas y franjas horarias, es decir, conductores, policías de tránsito, vendedores ambulantes y ciclistas, entre otros, sin dejar de lado a los menores de 5 y los mayores de 65 años, que son más vulnerables ante la exposición a estos contaminantes.

## Discusión

Se determinaron las zonas con mayores concentraciones de PM_25_, todas ubicadas en el suroccidente de la ciudad, principalmente en la localidad de Kennedy, tal como lo establecieron García, *et al.,* en un estudio que concluyó que la estación de Kennedy presentaba los mayores valores de PM_2,5_, con promedios anuales y mensuales en un rango de 30 a 45 µg/m^3^ entre ' el 2009 y el 2011 ^28^. No obstante, los promedios por franja registrados en esta estación en el presente estudio fueron de 33 µg/m^3^ (en el 2014) y de 30 µg/ m^3^ (en el 2015), lo que evidencia una disminución de las emisiones con el paso de los años. Además, en el estudio se encontró que las mayores concentraciones se daban de 6:00 a. m. a 12:00 m. y entre las 6:00 p. m. y las 0:00, es decir, las franjas b y d del presente trabajo, lo que coincide con las horas de mayor tráfico vehicular de la ciudad, así como el comienzo y el final de algunos procesos industriales.

Por otro lado, con base en los datos de 1997 al 2007, Gaitán, *et al.*^29^, establecieron que la zona con mayores concentraciones de PM_10_ era la localidad de Puente Aranda; la estación de monitoreo de esta localidad se encuentra entre la calle 13, la avenida 68 y la Avenida de Las Américas, lo que genera un punto de emisión relevante en esta zona. No obstante, los resultados del presente estudio evidenciaron que esta área sí presenta una gran influencia de las concentraciones registradas ya que, principalmente en las horas pico de la mañana, se ve afectada por la dispersión de los contaminantes del aire, aunque no es la que registra los mayores niveles de inmisión de la ciudad. En consecuencia, se podría afirmar que las diferentes políticas, regulaciones y mecanismos que se han planteado se han ejecutado hasta cierto punto y han permitido un comportamiento aceptable de los productos contaminantes en la zona ^30^^,^^31^.

Se sabe que una de las mayores fuentes de PM_25_ es la combustión que proviene de los vehículos, por lo tanto, el hecho de que las estaciones de monitoreo de Kennedy y Carvajal se ubiquen entre dos vías principales y de gran afluencia vehicular, como la Autopista Sur y la Avenida Boyacá, contribuye considerablemente en las emisiones de PM_2,5_. Sin embargo, estaciones como Ferias, que se encuentra muy cerca a' la calle 80, y la del Centro de Alto Rendimiento, ubicado entre la avenida 68 y la Avenida Carrera 30, no presentan promedios altos de PM_2,5_, tal vez porque no son zonas industriales y tienen algunas zonas verdes como el humedal Niza, cercano a la estación de Ferias, así como los parques Simón Bolívar y Distrital del Salitre, próximos a la estación de Alto Rendimiento, lo cual se asocia con los servicios ecosistémicos que ofrecen las zonas arbóreas urbanas: la regulación de la temperatura ambiente, el embellecimiento paisajístico, la reducción de emisiones de ruido, la atenuación de escorrentía de aguas pluviales y la mejora en la calidad del aire, que se evidencia en la disminución de buena parte de las PM_2,5_ mediante su incorporación en la cera de las hojas, mientras que parte del PM_10_ se resuspende de nuevo por la acción del viento y el resto va a los suelos por acción de la precipitación ^32^.

Los beneficios de la vegetación para la calidad del aire también se observan en las zonas nororiental y oriental de la ciudad, las cuales limitan con los cerros orientales, por lo que estaciones como las de Minambiente, Guaymaral, Usaquén e, incluso la de San Cristóbal, próximas a ejes viales importantes como la Autopista Norte, la carrera 7^a^ y la 10^a^, registraron valores de PM_25_ bajos y, en algunas horas del día, cumplieron con los estándares de las guías de la OMS, precisamente por su ubicación cercana a los cerros, con lo cual se benefician de los servicios que brinda su cobertura vegetal, además de hacer parte de la estructura ecológica principal del Distrito ^33^.

Se evidenció que los mayores niveles de PM_25_ siempre se concentran y dispersan en la zona suroccidental de la ciudad, que tiene una intensa actividad industrial y un aporte importante de fuentes móviles en vías principales que aumentan las emisiones de partículas finas de PM_2,5_ debido a los procesos de combustión. Esta zona de la capital limita con municipios como Soacha, Mosquera, Funza y Cota, en los cuales también hay parques industriales que generan emisiones de partículas en suspensión y son puntos de llegada de vehículos de carga pesada que producen abundantes emisiones. Asimismo, la dinámica de los vientos es un factor importante. Según el informe anual de la Red de Monitoreo de Calidad del Aire de Bogotá en el 2015, hay una acentuada tendencia a registrar vientos con bajas velocidades en el sur y el nororiente, lo que puede influir en las bajas concentraciones de PM_25_ en ciertas franjas horarias; sin embargo, los vientos con dirección de oriente a occidente predominan, lo que es una variable de gran influencia en la dispersión de PM_25_ en esta zona de la ciudad ^34^.

En la caracterización socioeconómica del 2016, la Secretaría Distrital de Planeación de Bogotá reportó que las localidades con mayor población eran Suba y Kennedy, lo que queda confirmado en los mapas de densidad poblacional de la Infraestructura de Datos Espaciales para el Distrito Capital, en los que la zona occidental de la ciudad aparece como una de las más pobladas, específicamente las localidades de Bosa, Kennedy, Fontibón, Engativá y Suba ^35^.

Por lo tanto, un gran número de personas se ven afectadas por las concentraciones de PM_2,5_, principalmente los niños de la primera infancia (0 a 5 años), las mujeres en estado de embarazo y las personas mayores de 65 años que, por su edad, e incluso su estado de salud, permanecen en sus hogares en ese sector, con una mayor exposición a los contaminantes. Según la Secretaría Distrital de Planeación, en el 2014 se registraron, aproximadamente, 88.221 personas en la localidad de Bosa, 150.599 en Kennedy, 52.826 en Fontibón, 131.750 en Engativá y 162.919 en Suba pertenecientes a los grupos etarios de menores de 5 años y mayores de 65 ^36^, lo cual representa un gran número de habitantes expuestos de forma puntual y permanente a las emisiones de PM_2,5_.

El análisis espacial en el presente estudio permitió visualizar la distribución de las concentraciones de PM_2,5_ en la ciudad en diferentes franjas horarias y clasificar los niveles según los valores de referencia de la OMS, lo que lo hace innovador y útil para relacionar de manera espacio-temporal cierto tipo de datos con localizaciones o ubicaciones para responder inquietudes y dar solución a algunos problemas ambientales ^15^, además de permitir la comparación con otros estudios que utilizan los valores de referencia de la OMS.

Si bien es cierto que entidades públicas como la Secretaría de Ambiente y la de Salud de Bogotá analizan la distribución de contaminantes, los métodos que emplean son los de distancia inversa ponderada, que solamente tienen en cuenta el dato registrado en una estación de monitoreo, en tanto que el procesamiento de datos en el presente estudio incluyó un análisis de sus propiedades estadísticas, lo que permitió evaluar la distribución del contaminante en varias direcciones y obtener mapas de predicción que permiten evaluar la variabilidad espacial del dato medido ^37^.

En varios estudios en el mundo se han empleado los análisis geoestadísticos para contrastar las concentraciones anuales de contaminantes en países desarrollados y en desarrollo. En el 2014, la OMS elaboró un mapa global de la exposición a PM_2,5_ utilizando los datos de las redes de monitoreo de calidad del aire de las regiones de África, Latinoamérica, América, el Mediterráneo oriental, Europa, sudoeste de Asia y Pacífico occidental, y encontró que más del 92 % de la población mundial está expuesta a niveles de PM_25_ superiores a los valores de las guías ^38^. En Taiwán se registraron concentraciones de PM_25_ que superan los niveles de la OMS, especialmente en las franjas horarias de las 8:00 a las 9:00 h y de las 14:00 a las 15:00 h, es decir, las de mayor tráfico vehicular ^39^. En Cusco (Perú) se han medido altas concentraciones en las horas de la mañana, con valores máximos de 35,4 µg/m^3^ en el 2010 ^40^, y en ciudades de Italia como Milán, Roma y Génova, muy pobladas y con una gran actividad industrial, también se han registrado altos niveles de PM_25_^41^. Esto es similar a lo encontrado en el presente estudio, respaldado por los registros horarios de la Red de Monitoreo de Calidad del Aire de Bogotá, y ratifica la problemática mundial de la contaminación del aire que afecta a los países latinoamericanos como Colombia.

Entre las fortalezas del presente estudio, cabe mencionar que es el primero en Bogotá con un diseño ecológico del análisis espacial mediante técnicas geoestadísticas y clasificación de las mediciones de PM_2,5_ según los valores diarios y anuales de referencia para enfermedades cardiopulmonares de la OMS en un periodo de más de un año. Además, se hizo una categorización por franjas horarias, lo que es un gran aporte para conocer las características de la exposición a PM_2,5_ en la ciudad en diferentes momentos del día.

Es importante señalar que el diseño utilizado no infiere una relación o causalidad entre la exposición y las enfermedades, pero sí permite plantear hipótesis o calcular un posible impacto en la población, al clasificar las concentraciones registradas según los valores de referencia de la OMS, los cuales reflejan la asociación entre la exposición a las PM_2,5_ y la mortalidad cardiopulmonar según diversos estudios epidemiológicos ^27^.

Otra de las fortalezas del estudio fue el uso de la relación de PM_25_/PM_10_ para complementar los datos de las PM_2,5_, lo cual contribuyó a fortalecer la calidad y solidez de la información, cubriendo los vacíos en algunas estaciones y logrando una mayor representatividad en el tiempo, como se aprecia en los mapas elaborados.

Entre las debilidades del estudio, debe mencionarse que solo se trabajó con una serie de dos años, aunque inicialmente se contaba con información desde el 2008 hasta el 2015. No obstante, al depurar los datos pertinentes, se encontró que no todas las estaciones monitoreaban las PM_25_ y la Red de Monitoreo de Calidad del Aire de Bogotá no tenía una buena cobertura de la ciudad, por lo cual se limitó el análisis a dos años y 10 estaciones que reportan concentraciones de los dos contaminantes. Así, se tuvo una mayor cobertura de la ciudad y registros horarios de más del 75 %.

Otra debilidad del estudio fue no contar con información de la población residente y flotante en el área de influencia de las estaciones de la Red de Monitoreo, ya que no se establece su radio estándar de cobertura debido a la topografía urbana de las zonas. Sin embargo, mediante el análisis geoestadístico se evaluó la distribución del PM_2,5_ en diferentes direcciones, lo que se tradujo en mapas de predicción con los que se estimó la variabilidad espacial del dato medido.

Dadas las diversas actividades comerciales e industriales de Bogotá, los mapas permitieron evidenciar que las localidades más afectadas eran Fontibón, Bosa, Puente Aranda, Tunjuelito, Kennedy y parte de Suba, Engativá, Teusaquillo, Ciudad Bolívar, Rafael Uribe Uribe y Antonio Nariño. Se resalta que Kennedy fue la más afectada en todas las franjas horarias al registrar las mayores concentraciones de PM_2,5_ asociadas con enfermedades cardiopulmonares debidas a la exposición crónica o aguda a estos contaminantes según los valores de referencia de la calidad del aire de la OMS. En esta localidad, las unidades de planeación zonal más afectadas fueron Castilla, Patio Bonito, Las Margaritas, Corabastos, Kennedy Central, Américas, Gran Britalia, Carvajal y Timiza, con concentraciones promedio en un rango entre 27 y 32 µg/m^3^ que, comparados con los valores sugeridos por la OMS en un promedio de 24 horas, se ubican en el objetivo intermedio 3, es decir, aquellos que incrementan en 1,2 % la mortalidad a corto plazo con relación a los de las guías, que son los más bajos asociados con los coeficientes de riesgo publicados en estudios multicéntricos y metaanálisis. Estas concentraciones comparadas con el valor promedio anual establecido por la OMS corresponden al objetivo intermedio 2, es decir, el que aumenta el riesgo de mortalidad en 9 % teniendo como referencia los niveles más bajos con los cuales se ha demostrado que la mortalidad total, cardiopulmonar y por cáncer de pulmón aumenta en respuesta a la exposición prolongada a las PM_2,5_^27^.

En este contexto, los menores de 5 años y los adultos mayores de 65, que representan el 10,2 y el 8,7 % del total de la población de la localidad, respectivamente ^42^, se verían afectados la mayor parte del día.

Por otro lado, hay zonas de la ciudad con bajas concentraciones que, comparadas con el valor diario sugerido por la OMS, cumplen con las guías (25 µg/m^3^). Sin embargo, el panorama con el valor anual es diferente, ya que solo en las franjas horarias a y c (de las 0:00 a las 6:00 h y de las 12:00 a las 18:00 h) se cumple con los valores de las guías (10 µg/m^3^) en una parte de la zona oriental que abarca parte de las localidades de Usaquén, Chapinero, Santa Fe y San Cristóbal, es decir, los mínimos en los que se observan efectos significativos en la salud humana, sin descartar que por debajo de ellos haya efectos adversos para la salud ^27^.

Por último, aunque este trabajo no permite establecer una relación causal, sí brinda las bases para el desarrollo de estudios de series de tiempo que permitan evaluar la relación entre la exposición a PM_2,5_ y la mortalidad por enfermedades cardiopulmonares en Bogotá, con lo que se ampliaría el panorama reflejado en otros estudios, como los de Ciudad de México, Santiago de Chile y la misma Bogotá, en los cuales se demostró que los valores de PM_10_ de referencia según la OMS se superaron entre tres y cuatro veces, con el consecuente incremento del riesgo de morbilidad y mortalidad en temporadas invernales o frías ^43^.

Específicamente en Bogotá, se estimó que la exposición a niveles elevados de PM_10_ estaba asociada con el incremento de la mortalidad respiratoria y cardiovascular, especialmente en personas provenientes de estratos socioeconómicos bajos (44), y se ratificaron los datos recientes sobre la relación entre la exposición a PM_2,5_ y la morbilidad cardiopulmonar en diferentes ciudades de Colombia ^45^^,^^46^.

El presente estudio aporta a la implementación de estrategias y planes como el plan decenal de descontaminación del aire en Bogotá, 2010-2020, la política distrital de salud ambiental y la dimensión de salud ambiental del Plan Decenal de Salud Pública, 2012-2021 , contribuyendo así a mejorar la calidad de vida y la salud de los habitantes de Bogotá.
